# Blood flow restriction with different load levels in patients with knee osteoarthritis: protocol of a randomized controlled trial

**DOI:** 10.1186/s13063-022-05998-3

**Published:** 2022-01-15

**Authors:** Roger Andrey Carvalho Jardim, Tamara Silva de Sousa, Wueyla Nicoly Nascimento dos Santos, Areolino Pena Matos, Natália Camargo Rodrigues Iosimuta

**Affiliations:** 1 Postgraduate Program of Health Sciences, Department of Biological Sciences and Health, Federal University of Amapá - UNIFAP, Macapá, AP Brazil; 2 Physical Therapy Course, Department of Biological Sciences and Health, Federal University of Amapá - UNIFAP, Macapá, AP Brazil

**Keywords:** Osteoarthritis of knee, Blood flow restriction training, Kaatsu training, Muscle strength, Resistance training

## Abstract

**Background:**

The effectiveness of blood flow restriction training (BFR) in elderly with knee osteoarthritis (OA) is comparable to performing high-intensity protocols (70 to 80% of 1 RM [repetition maximum]) that are known to be effective for improving the muscle strength of knee extensors, with the advantage of generating less particular rating of perceived exertion and pain immediately after training. However, despite being a promising alternative, little is known about the best way to apply the BFR, such as level of pressure and combination or not with other therapeutic modalities. The purpose of this study is to evaluate whether different levels of blood flow restriction with low load (BFR + LL) and no load (BFR + rest) are non-inferior to high-intensity resistance exercise (HIRE+BFRplacebo) for pain reduction in patients with knee OA.

**Methods/design:**

This clinical trial is a non-inferiority, five-arm, randomized, active-controlled, single trial which will be carried out in 165 patients of both sexes with knee OA, aged 50 years and older. Participants will be randomly allocated into 5 exercise groups (40% of BFR + LL; 80% of BFR + LL; 40% of BFR + rest; 80% BFR + rest, and HIRE+BFR placebo). A mixed linear model will be used to examine the effect of group-by-time interaction on pain intensity on the WOMAC subscale (primary outcome) and on disease severity, physical functional data, balance data, quality of life, global perceived effect scale, and muscle strength (secondary outcomes). Participants will be analyzed for intention-to-treat, and the statistical assessor blinded to the groups. The collection of outcomes 72 h after completion of the 16 weeks of interventions will be the primary measurement point. Follow-up secondary timepoints will be collected at 20, 28, 40, 52, and 64 weeks after the end of interventions, except for pain during the training, which will be measured immediately at the end of each session. Only the comparison of the primary outcome between the HIRE group with each BFR group will be analyzed in the non-inferiority framework, the other comparisons between the BFR groups for the primary outcome, and all secondary outcomes will be interpreted in the superiority framework.

**Discussion:**

The results of this clinical trial can point out more clearly to ways to optimize the BFR training with the minimum of pain immediately after training, which will allow the offer of an effective and more adherent strengthening training to patients with knee OA.

**Trial registration:**

*Registro Brasileiro de Ensaios Clínicos*, RBR-93rx9q. Registered on 23 July 2020. Version 1.0.

**Supplementary Information:**

The online version contains supplementary material available at 10.1186/s13063-022-05998-3.

## Background

Osteoarthritis (OA) is the most common form of arthritis in the world with an increasing number of cases, resulting from the increase of aging and obesity in the world population [1, 2]. In 2017, there were 307 million people diagnosed with OA in the world [3], resulting in costs ranging from 1 to 2.5% in the gross domestic product in developed countries [4].

The articular cartilage and subchondral bone of a synovial joint is the main region affected in OA, often resulting in pain, physical disability, and quality of life impairment [5]. Worldwide, the most affected joint in approximately 75% of all OA cases is the knee joint [1]. The risk factors for the development of knee OA include advanced age, female gender, obesity, previous joint injury, weakness of the knee flexor and extensor muscles, profession, and genetics [6, 7]. Regarding advanced age, the elderly population is especially susceptible to OA because of the loss of global muscle mass [8], gradual reduction of articular cartilage [9], alteration of bone density [10], and reduction in the level of physical activity [2, 11].

For the treatment of OA, studies show that strengthening knee extensor muscles is a priority in the management of knee OA [12] with a well-established hypertrophy training protocol for the elderly population [13].

According to current guidelines, physical exercise is recommended to treat knee OA regardless of disease severity, pain levels, and functional status [12, 14–16]. Besides, the recommendations of the World Health Organization’s guidelines for physical activity shows that the elderly have to perform at least 150 min of moderate to vigorous physical activity per week in sessions of at least 10 min. However, only 13% of older people with knee OA satisfactorily reach that target [17, 18]. Unfortunately, high-intensity resistance exercise (HIRE) demands the use of high loads that can worsen the pain and cause swelling as well as inflammation in individuals with knee OA [19]. Thus, with the appearance of these symptoms, there is a reduced adherence to exercises [20].

Because of the low adherence of patients with knee OA to HIRE, new training modalities for improving muscle strength have been investigated recently, such as aquatic exercises [21], neuromuscular [22], high speed [23], and blood flow restriction training (BFR) [24].

The BFR has shown promising results in musculoskeletal rehabilitation. It consists of a momentary and controlled mechanical compression of the proximal segment of the limb [25]. The most accepted mechanisms that can explain the development of muscle hypertrophy in BFR is the accumulation of metabolites around the trained muscle as an adaptive response to local hypoxemia [26, 27].

The effectiveness of BFR in patients with knee OA is comparable to traditional protocols for gaining muscle strength in knee muscle extensors [28–30]. However, there is the advantage of generating less joint particular rating of perceived exertion and pain immediately after training [31] and providing results in 6 weeks [32–34] compared to 8 weeks, the training duration commonly needed in HIRE [35].

Despite being a promising alternative, there is still no ideal protocol for applying the BFR in patients with knee OA. Thus, there is still lack of information about the most efficient and safety levels of pressure applied by the cuff to restrict blood flow and on the combination or not of the BFR with other therapeutic modalities [36]. Positive results for muscle strength improvement have been found using the BFR with 40 to 90% of the total arterial limb occlusion pressure (LOP) [37], associated with resistance exercises using low loads that vary between 10 and 30% of 1 RM [25]. Yet, no study has assessed whether BFR at rest could also promote similar gains in muscle strength for the knee OA treatment, which theoretically would increase adherence to knee OA treatment by patients, for minimizing the physical discomfort commonly presented in high and moderate-intensity exercises [31].

Therefore, the aim of this research is to assess whether different protocols of BFR are non-inferior to HIRE with placebo BFR for pain reduction in patients with knee OA.

## Methods/design

### Study design

**Fig. 1:**
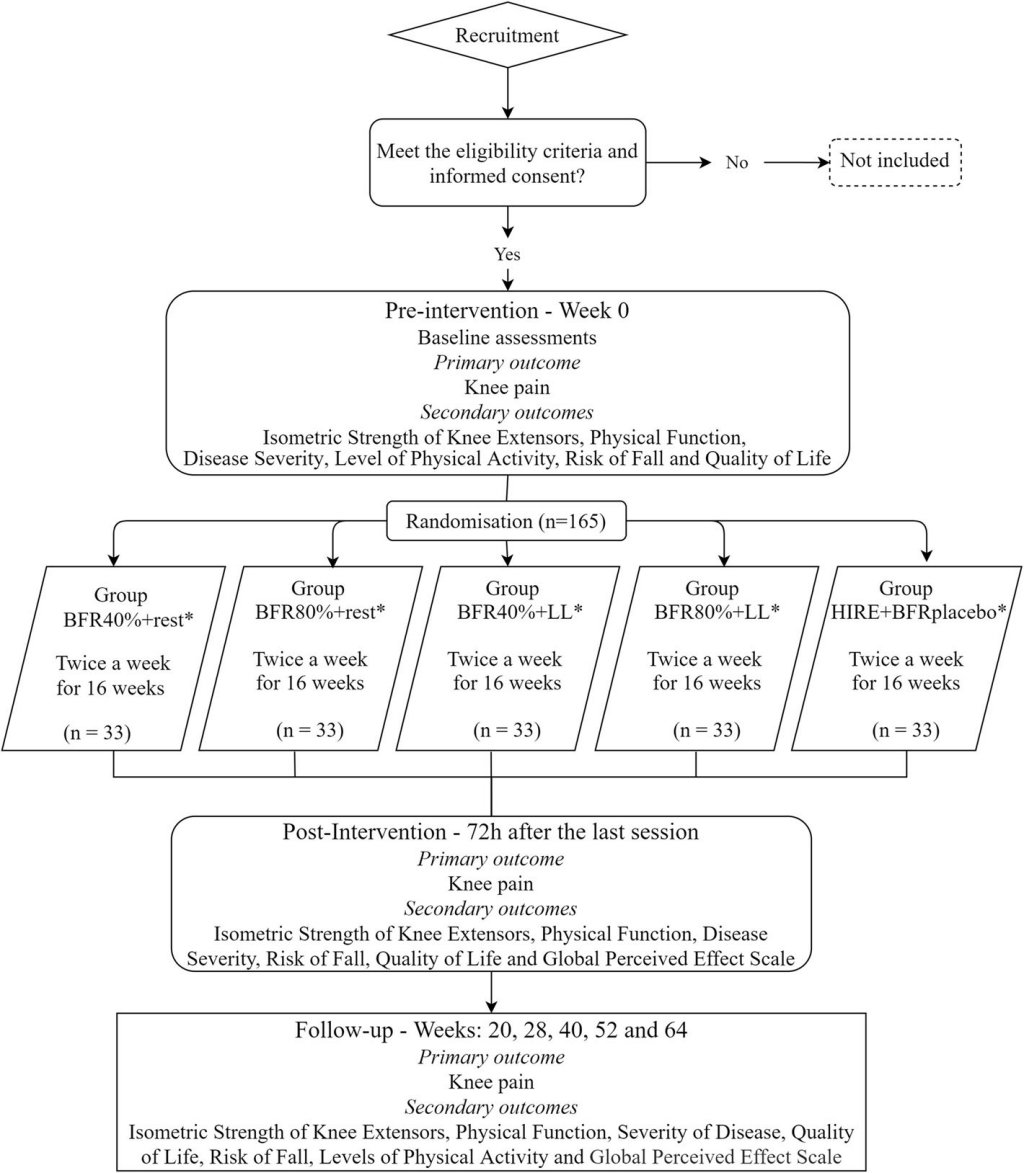
Consolidated Standard of Reporting Trials flow chart illustrating the process of the study. BFR40% + rest: restriction of 40% of blood flow without load. BFR80% + rest: 80% restriction of blood flow without load. BFR40% + LL: 40% restriction of blood flow with low load. BFR80% + LL: 80% restriction of blood flow with low load. HIRE+BFRplacebo: high-intensity resistance exercise with 80% of a repetition maximum and blood flow restriction placebo. *Immediately upon the conclusion of the sessions, the results of the knee pain will be recorded

This is a clinical trial of non-inferiority, randomized, active-controlled, single-center trial, with five parallel groups and concealed allocation. Four groups will use BFR with different protocols, and one group will receive HIRE associated with placebo BFR. The intervention groups will be performed for 16 weeks and after this, a follow-up will take place in 6 timepoints, as shown in Fig. 1. The trial was registered at the *Registro Brasileiro de Ensaios Clínicos* (RBR-93rx9q).

**Fig. 2:**
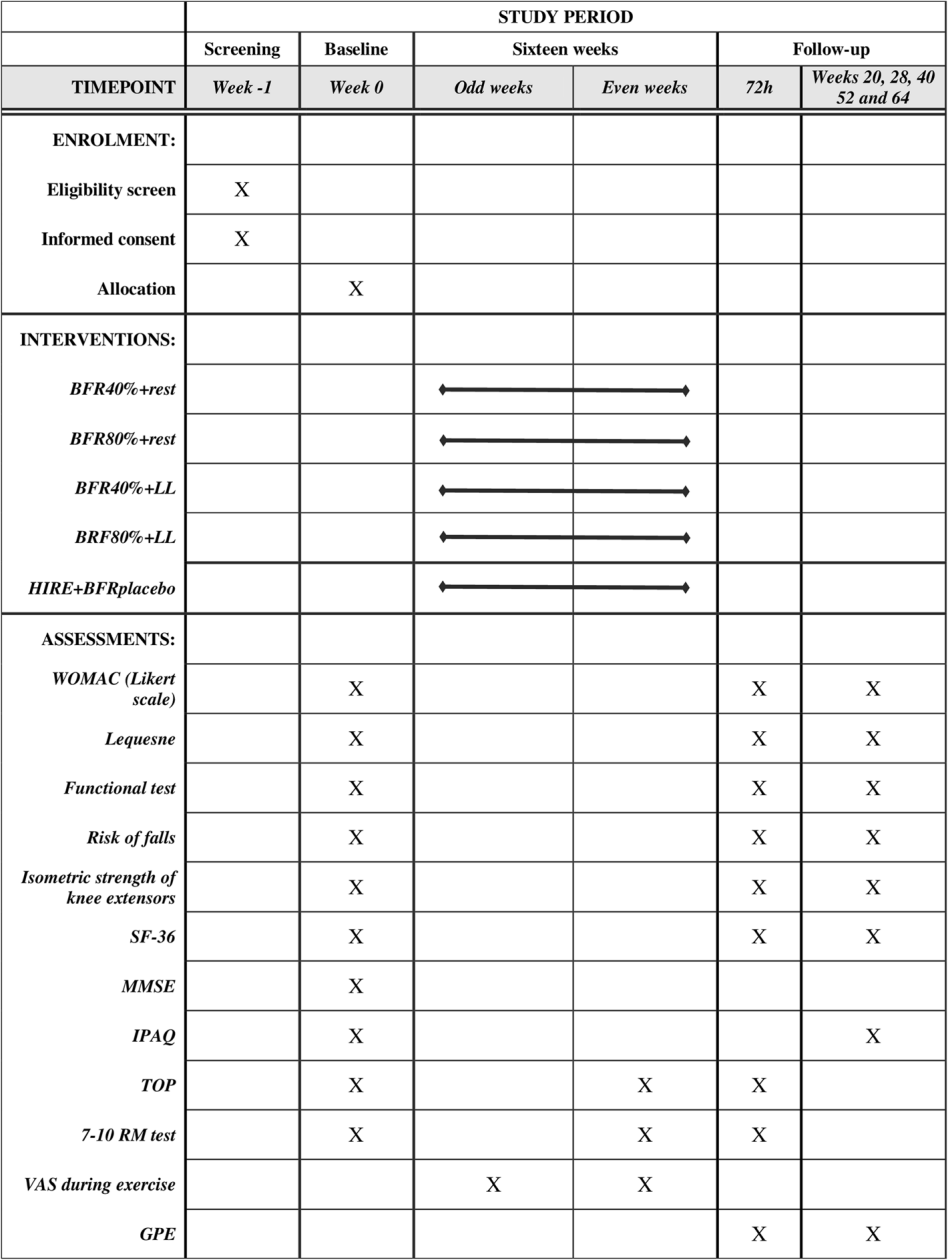
Study design schedule in accordance with the Standard Protocol Items: Recommendations for Interventional Trials. BFR40% + rest: restriction of 40% of blood flow without load. BFR80% + rest: 80% restriction of blood flow without load. BFR40% + LL: 40% restriction of blood flow with low load. BFR80% + LL: 80% restriction of blood flow with low load. HIRE+BFRplacebo: high-intensity resistance exercise with 80% of a repetition maximum and blood flow restriction placebo.WOMAC: Western Ontario and McMaster Universities. SF 36: Short-Form Health Survey. MMSE: Mini-Mental State Exam. IPAQ: International Physical Activity Questionnaire. TOP: Total Arterial Limb Occlusive Pressure. RM: Repetition Maximum. VAS: visual analog scale for pain. GPE: Global Perceived Effect

All assessments and the protocol will be carried out in one of the laboratories of the Physiotherapy Course, Federal University of Amapá-AP, Brazil. Participants with knee OA will be recruited from the community in Macapá-AP, Brazil. The invitation will be made through electronic media, folders, personal invitation, and telephone contact. Outcome measures will be performed according to the sequence and schedule shown in Fig. 2.

The study protocol was developed according to the guidelines of the standard items of the protocol: Recommendations for interventional trials (SPIRIT) guidelines (Additional file 1) [38] and Consolidated Standards of Reporting Trials (CONSORT) guidelines [39].

### Eligibility criteria

In total, 165 participants will be included if:

1. Over 50 years old;
2. Diagnosed with bilateral or unilateral knee OA according to the American College of Rheumatology criteria;
3. Moderate to very severe (scores between 5 and 13 on the Lequesne Questionnaire);
4. Minimum score of 24 points on the Mini-Mental State Examination and those who sign the Informed Consent Form (ICF).

Patients will be excluded if they have:

1. History of surgery or any invasive procedure on the knee(s) with OA;
2. Undergone a physical therapy or muscle strengthening program for lower limb in the past 3 months;
3. Planned events, such as knee replacement or travel, which may interfere with the adherence of individuals during the trial period;
4. History of acute myocardial infarction and/or stroke;
5. History of peripheral arterial disease or deep vein thrombosis;
6. History of cancer that has generated limitations or restrictions to physical exercise;
7. Decompensated systemic blood pressure without medical supervision;
8. Changes in the dose or type of anti-inflammatory or analgesic drugs in the last 3 months.

One of the researchers (WNNS) will check the volunteers’ eligibility criteria and apply the study ICF. Participants who discontinue their attendance in the study will be invited to participate in the assessments during the follow-up period (Fig. 2).

Thus, all individuals will be included in the analysis as for their intention to treat. Participants who discontinue the proposed treatment and the assessments will be considered as subject loss to follow-up, which should not exceed 15% of the original sample size. The therapists who will be responsible for the intervention groups will receive specific training for the treatment protocol.

### Randomization

Participants will be randomly allocated in the equal ratio between groups (1:1:1:1:1) using the generator available at www.randomization.com by a researcher (APM) who will not be aware of the research group participants and evaluations. Balanced permutations in blocks will be used in relation to the presence of unilateral or bilateral knee OA.

The allocation of participants will be hidden in opaque numbered and sequentially sealed envelopes, prepared before the study by a researcher (APM) who did not participate in the recruitment and assignment of the groups. The corresponding envelopes will be opened once the participants enrolled have completed all baseline assessments.

The researcher responsible for evaluations (TSS) and data analysis (NCRI) will be blind to the groups of participants. The order of the evaluations will follow a predetermined sequence that will try to reduce the interference of the physical fatigue of a test in its successor:

- Set 1: WOMAC, MMSE, and LOP;
- Set 2: IPAQ, Time Get Up and Go Test (TUG) and 30-second Chair Stand Test (30-sCST);
- Set 3: SF-36, Lequesne and isometric strength evaluation of the knee extensors;
- Set 4: Clinical Test of Sensory Interaction and Modified Balance, 40-m Fast Paced Walk Test (40mFPWT), Demographic Data and 7-10 RM test.

### Intervention

The intervention protocol will take place in one of the laboratories of the Physiotherapy Course, Federal University of Amapá-AP. The researcher (RACJ), who is a physiotherapist and specialized in BFR and HIRE techniques, will be responsible for the intervention groups.

In a systematic review, Borde, Hortobágyi, and Granacher [13] identified the main parameters of resistance training capable of increasing muscle strength in the elderly. In accordance with this finding, the interventions will take place twice a week with a 40-min duration, for 16 weeks, totalizing 32 sessions (Fig. 2). The reference for the volume of exercises will be provided by the HIRE group, which will perform 3 sets of 8 repetitions at 80% of 1 RM load, with 60 s of rest between sets [13]. The total volume of the exercises will be calculated by multiplying the weight, repetitions, and series [19]. For groups with restricted blood flow and low load with 40% of LOP (BFR40% + LL) and with 80% of occlusion (BFR80% + LL), the load will be fixed at 30% of 1 RM [40], and the maximum number of repetitions will be 15 repetitions per series, increasing the serial number according to the volume equivalence used in the HIRE group.

For groups with BFR without load with 40% of LOP (BFR40% + rest) and with 80% of occlusion (BFR80% + rest), the reference for the restriction time will be the average restriction time of the groups with low load, with a minimum of 10 min per session [24]. The choice for BFR pressures is based on studies that have demonstrated a significant decrease in blood flow from 40% of the LOP [41], with no additional improvement until the relative pressure of 80% [42]. The summary of interventions is compiled in Table 1.

**Table 1:** Blood flow restriction and exercise intervention protocol

	BFR40% + rest*	BFR80% + rest*	BFR40% + LL*	BFR80% + LL*	HIRE + placebo*
BFR application	•40% of the total blood occlusion pressure, bilateral concomitantly. • Time: determined by the average of the BFR + LL group, with a minimum time of 10 min, distributed in series of maximum 5 min of consecutive restriction, and 60 s of rest between series.	•80% of the total blood occlusion pressure, bilateral concomitantly. • Time: determined by the average of the BFR + LL group, with a minimum time of 10 min, distributed in series of maximum 5 min of consecutive restriction, and 60 s of rest between series.	•40% of the total blood occlusion pressure, bilateral concomitantly. •Time: used throughout the exercise period and deflated during rest between sets.	•80% of the total blood occlusion pressure, bilateral concomitantly. •Time: used throughout the exercise period and deflated during rest between sets.	•10% of the total blood occlusion pressure (BFR placebo), bilateral concomitantly. •Time: used throughout the exercise period and deflated during rest between sets.
Exercise application	• Rest in a sitting position.	• Rest in a sitting position.	• Mode: bilateral knee extension between 90° and 45°. • Load: 30% of 1 RM. • Series: determined by the equivalence of the training volume with the HIRE+placebo group with a maximum number of 15 repetitions per series. •Rest: 60s between sets.	• Mode: bilateral knee extension between 90° and 45°. • Load: 30% of 1 RM. • Series: determined by the equivalence of the training volume with the HIRE+placebo group with a maximum number of 15 repetitions per series. •Rest: 60s between sets.	• Mode: bilateral knee extension between 90° and 45°. • Load: 80% of 1 RM. • Series: 3 series of 8 repetitions. • Rest: 60s between sets.

*BFR* blood flow restriction, *LL* low load, *HIRE* high-intensity resistance exercise, *RM* repetition maximum. *Intervention applied twice a week for 16 consecutive weeks

The 30% loads of 1 RM for the BFR40% + LL and BFR80% + LL groups, and 80% for the HIRE+BFRplacebo group, will be estimated (1 RM estimate) from the maximum load that can be exceeded in 7 to 10 repetitions (7 to 10 RM test) [43], based on the Brzycki equation (*W* / (1.0278 − 0.0278 × *R*), where *W* refers to the weight used in repetitions until failure and *R* refers to repetitions for failure [44]. The load will be readjusted every 2 weeks with an interval of 2 to 4 days after the last treatment session to prevent residual exercise fatigue from interfering with the tests from 7 to 10 RM. As for the 1 RM test, the 7–10 RM test has the advantage of minimizing the effect of pain on maximum strength generation [43].

The 7–10 RM estimation session and all sessions for all groups will start with 5 min of warm-up on a bicycle, before any intervention. The goal for the warm-up period is that participants exercise less than 11 (light) on Borg’s perceived effort rating scale, scored from 6 to 20 [45]. For groups with exercises with load (BFR40% + LL, BFR80% + LL, and HIRE+BFRplacebo), strengthening will be performed through bilateral knee extension exercise (angle between 90° and 45° of knee flexion) on the chair extensor [46], based on previous protocols for protecting the patellofemoral joint during exercises [47]. The training parameters will be adjusted having the HIRE group as a reference, so that there is no difference in the training volume between groups with exercises, as shown in Table 1.

Participants will be warned that knee pain or discomfort during exercise is normal and that it does not necessarily cause joint damage [48]. The load will be reduced by 20% compared to the 1 RM estimate if the pain prevents the volunteers from completing the exercise [49].

### Blood flow restriction

The LOP will be estimated individually to generate a similar metabolic stimulus among the participants [42]. After 10 min of rest in a climatized room (between 23° and 25 °C), the LOP will be determined with the volunteers seated and with the upper limbs relaxed at the side of the body. The transducer (5 to 10 MHZ) of a portable vascular Doppler (DV 610, Medmega) will be positioned at the ankle at a medium distance between the medial malleolus and the calcaneus tendon to capture the auscultatory signal from the posterior tibial artery [40]. A 13.5-cm-wide pneumatic cuff will be positioned at the proximal end of the thigh and inflated until the auscultatory signal ceases, indicating occlusion of the artery [50, 51].

In the HIRE+placebo group, a minimum restriction pressure of 10% of the LOP will be applied, an extremely low dose that does not influence the volume of the exercises performed [52]. The same cuff used in the assessment of LOP will be inflated in the proximal end of the thigh with the participants seated for rest in the unloaded groups, and in knee extension exercises for the other groups. The cuff will be deflated during the rest interval between sets. Fluctuations in the prescribed pressure (10%, 50%, and 80% of LOP) will be monitored and regulated by the therapist. The number of repetitions completed in each series will be monitored to verify that the total proposed volume has been reached, then the cuff will be deflated.

During the evaluations and exercises, the position of the cuff will be adjusted so that the non-insufflated portion of the equipment is in the lateral region of the thigh, away from the femoral artery located in the medial compartment of the leg. This way, a smaller LOP is necessary [53], except for the HIRE+placebo group, in which the area without cuff insufflation will be positioned over the femoral artery so that there is the least possible blood restriction.

### Criteria for discontinuing or modifying allocated interventions

The criteria for discontinuing or modifying intervention allocation of a particular participant from the study will be as follows: occurrence of severe adverse events or threat to life, as specified in the “Adverse event reporting and harms” section or if the participant has any health condition that forbids him from receiving study interventions.

If any participant discontinues the study or changes allocation, data will be analyzed considering the initial allocation of the participant as he/she was initially randomized. Once the interventions delivered (BFR and HIRE) are known to be safe [54], no interim analyses are planned for participants who discontinue before the study timeline, nor for external committees for data monitoring.

In addition, although the BFR and HIRE are known to be safe, adverse events, such as harm, could happen. If any participant gets harmed from interventions, the authors guarantee proper care by medical and/or physiotherapy professionals at the Federal University of Amapá.

### Plans to promote participant retention and complete follow-up

For promoting participant retention and complete follow-up, the plans of the authors are as follows: make an understandable and as short as possible ICF; answer all questions from participants during ICF discussion and explain the importance of their participation, also clarifying and minimizing any concern from participants; ask about the participant’s expectations and aligned them with the aim of the study; send reminders for upcoming visits and follow-up by mail and telephone numbers of participants and/or their closest family members; accommodate the schedule as much as possible; provide a comfortable and patient-friendly environment; and show participants authors’ appreciation and recognition of their collaboration in the trial.

### Relevant concomitant care permitted or prohibited during the trial

All subjects included will be instructed not to change the current dosage of their anti-inflammatory and analgesics and will be encouraged to report any medical or pharmacological interventions received during the study. In addition, participants will be discouraged to start regular exercises and any other treatment during the interventions of the study and should inform the researchers if these recommendations are broken. The volunteers will be reevaluated at the end of the 16 weeks of treatment, and then they will be instructed to practice the regular exercises of their choice.

### Masking/blinding

The participants will not be informed about which group they belong to and will be instructed not to talk about their experience during the exercise in case they accidentally meet other participants. In addition to the placebo for the HIRE group, all participants will be informed that BFR is effective in increasing muscle strength and reducing knee pain.

The interventions, evaluations, randomization, and data statistical analyses will be carried out by different collaborators who will not share information about the research, making it difficult to influence the data assessed in different phases of the study. Despite these procedures, the characteristics of the interventions do not allow for blinding of participants, outcome assessors (self-reported), and therapists.

### Cognitive state

The assessment of cognitive status will be carried out through the Mini-Mental State Examination (MMSE) [55], an instrument used for tracking dementia and assessing cognitive function [56]. The translated and validated version in Brazil was proposed by Bertolucci et al. [57] and Almeida [58]. Scores range from 0 to 30 points, with the cut-off point for cognitive decline taking into account the respondent’s level of education, corresponding to the score: ≤ 13 points for illiterates, ≤ 18 points for those with 1 to 11 years of schooling, and ≤ 26 points for schooling over 11 years [57]. Possible cognitive decline will imply in participant exclusion.

### Primary outcome

The Western Ontario McMaster University Osteoarthritis Index (WOMAC) pain score is the primary outcome. WOMAC is a disease-specific quality of life questionnaire for use in osteoarthritis clinical trials [59]. The WOMAC pain subscale has 5 items, with the rating scale ranging from 0 (none) to 4 (extreme), with higher scores indicating worse painful conditions [60].

### Secondary outcomes

#### Evaluation of the severity of osteoarthritis

The Lequesne Algofunctional Index and the WOMAC are commonly used instruments to assess pain, physical disability, and disease severity in patients diagnosed with OA [61]. Studies previously conducted show that WOMAC and the Lequesne Functional Something Index have a good correlation. Bellamy et al. [60] demonstrated that the questionnaires show similar results when used to record improvement of patients with OA submitted to medication.

According to Samuel and Kanimozhi [59] when analyzing the results used in the diagnosis, prognosis, and rehabilitation of patients with knee OA, the WOMAC, and the Lequesne Algofunctional Index in the pain subscale shows reliability and a good correlation of results. However, although both address the severity of OA and the physical function of these patients, the questionnaires are not a substitute for assessing the symptoms and disabilities of these cases [62].

#### Functional assessment and risk of falls

The Time Get Up and Go Test (TUG), 30-second Chair Stand Test (30-sCST), and 40-m Fast Paced Walk Test (40mFPWT) are tests recommended by the Osteoarthritis Research Society International (OARSI), considered as a basic set of functional tests for assessing physical performance in patients with knee OA [63]. All three tests (TUG, 30-sCST, and 40mFPWT) are also used to assess balance and risk of falls [64, 65], mainly due to their having good to excellent correlation with poor balance and number of falls [66, 67]. The tests will be applied according to the protocol described by OARSI [63]. For TUG and 40mFPWT, tests completed in less time are associated with better fitness and lower risk of falling [64, 66]. For 30-sCST, a higher number of repetitions indicates better functional prognosis [67].

#### Clinical Test of Sensory Interaction and Balance Modified

The Clinical Test of Sensory Interaction and Balance Modified test is a balance test that assesses the visual, vestibular, and somatosensory influence of static balance in six sensory conditions: (1) standing, standing on a stable surface, eyes open; (2) on a stable surface, standing still, with eyes closed; (3) on an unstable surface, standing, standing with eyes open; (4) standing, standing on an unstable surface with eyes closed. Individuals will be instructed to remain for 30 s in each sensory condition, without taking any steps to compensate for the instability, and without moving the upper limbs, heels, and feet; if instability occurs before 30 s, the test is completed and considered as altered [68].

#### Level of physical activity

The level of physical activity will be verified using the Brazilian version of the International Physical Activity Questionnaire (IPAQ), long version. This questionnaire includes 27 questions about physical activities performed in a regular week, with vigorous, moderate, and light intensity, with a minimum duration of 10 continuous minutes, distributed in five domains: work, transportation, domestic activity, leisure/recreation and sitting. The level of physical activity will be classified continuously by calculating the estimated energy expenditure in METs (metabolic equivalent) [69].

#### Evaluation of muscle strength of knee extensors

The muscle strength of the knee extensors will be performed using the MicroFET2® manual dynamometer (Hogan Scientific - USA), which is a reliable and valid portable device for the evaluation of the isometric muscle strength and power of lower limbs [70]. The procedures for collecting the isometric strength of knee extensors will follow the steps of an already published study [71].

#### Quality of life assessment

The Brazilian version of the generic questionnaire of quality of life RAND 36-Item Short-Form Survey (SF-36) Version (1.0) [72] will be applied, which evaluates 8 domains, namely: functional capacity, limitation due to physical aspects, pain, general health, vitality, social aspects, emotional aspects, and mental health. The questionnaire score is given by domain and ranges from 0 to 100, in which the highest score reflects the best function.

#### Pain immediately after training

Knee joint pain during exercise will be assessed in all sessions (immediately after each set) by the pain VAS. Participants will be asked to report their knee pain score before starting interventions and immediately at the end of each session. The final score will be the difference between the first and second score.

#### Global Perceived Effect scale

For this research, the Global Perceived Effect Scale (GPE) scale was adapted to assess the patient perception of recovery from the first session and will be applied during all follow-up points. The guiding question was “Compared to the day you started the treatment, how do you describe your knee pain today?” It is a numerical scale of 11 points (− 5 to 5), with − 5 being much worse; 0 being no change; and 5 being complete recovery. The higher the score, the better the recovery from the condition [73].

### Sample size calculation

Sample size is based on detecting non-inferiority of the groups that will receive interventions with BFR compared to active placebo with exercise. For the change in pain on the WOMAC subscale (score 0–20), a non-inferiority margin (NIM) of 2.1 units was chosen as it is less than the minimal clinically important difference (MCID) reported as 2.4 points in the WOMAC questionnaire [74]. The margin of 2.1 units is the same of 11% of the total WOMAC pain subscale score, which is higher than the 8–9% commonly used in previous studies [75–77].

Assuming a standard deviation (SD) of 3.14 [78], 80% power at an alpha level of 5% to the primary measuring point 72 h after completion of interventions and a drop-out of 15%, we will need 33 participants per group, resulting in a total sample of 165 participants. The use of bilateral alpha value is in accordance with the recommendations of not ignoring superiority in non-inferiority trials [79, 80]. The sample size calculation was performed using the R software version 4.1.2.

### Statistical analysis

The statistical analysis will be conducted following the principles of intention-to-treat analysis. The normality distributions of the data will be assessed by visual inspection of histograms. In order to ensure data quality and data consistency between the source data and the data entered into the database, the data will be entered using the double entry method by two researchers (TSS and WNNS) independently into the database.

A linear mixed-effects model will be used to examine the between-group differences (BFR40% + rest, BFR80% + rest, BFR40% + LL, BFR80% + LL, and HIRE+placebo), time (baseline, 72 h, 20, 28, 40, 52, and 64 weeks), and group-time interaction in the primary outcome of pain intensity (measured using WOMAC subscale), and secondary outcomes of the disease severity, physical functional data, balance data, quality of life, pain during intervention, global perceived effect scale, and muscle strength. The 72-h follow-up will be the primary measurement point, and the other timepoints will be used to support the understanding of the effects of the interventions. Non-inferiority will be demonstrated if the lower limit of the 95% bilateral confidence intervals (CI) for the difference between the HIRE and each BFR group is above − 2.1 units for change in the WOMAC pain subscale. The comparison between the BFRs groups and the secondary results will be interpreted using the superiority framework since we have not pre-defined any NIMs for these outcomes.

Analyses will be adjusted for baseline demographic and clinical characteristics of age, sex, disease severity, BMI, and educational level, considering 80% power at an alpha level of 5%. The effect sizes (Cohen’s *d*) of the analyses will be presented with their respective 95% CIs. Chi-square tests will be used to test for possible differences in the adverse events observed between groups. All analyses will be calculated by one of the authors (NCRI) blinded to the allocation of groups, using tables with randomization codes. The statistical analysis will be conducted using the SPSS 25.0 software (SPSS, Chicago, IL, USA).

### Adverse event reporting and harms

During the trial, we will collect and record possible adverse events, describing the date of onset and date of resolution, evaluating event severity, investigating potential causal relationships with the intervention or with other suspect drugs, and assessing the potential effect of the event.

Some adverse events that may occur with BFR include rhabdomyolysis, subcutaneous hemorrhage, numbness, cold feeling, deep vein thrombosis, and itching [81, 82]. Severity must be defined according to the following criteria:

- Mild: Awareness of signs or symptoms, but easily tolerable;
- Moderate: enough discomfort to interfere with normal daily activities;
- Severe: inability to perform normal daily activities;
- Threat to life: immediate risk of death due to the reaction that occurred;

All adverse events will be tracked until the incident is resolved or the trial is completed.

## Discussion

This trial will investigate whether BFR at different pressure levels isolated or combined with low-intensity exercise shows similar results to HIRE in pain for individuals with knee OA. In addition, the disease severity, muscle performance, physical function, risk of falling, and quality of life will also be assessed.

Although previous studies have shown pain reduction with BFR training in individuals with OA, the protocol of BFR application is very heterogeneous. Haphazardly, BFR is estimated at 70% of the occlusion pressure [28], 200 mmHg [40], or through formulas [83]. In addition, its use is commonly combined with low-intensity resistance exercises. Then, the real effect of BFR in an elderly with chronic disease is unknown.

With this trial determining the best application of BFR, patients with knee OA, who do not adhere to traditional treatment protocols with physical exercise, will experience a new and less uncomfortable modality [40, 83]. If BFR proves to be an effective alternative for the treatment of knee OA, resources used for surgeries and care resulting from the worsening of the disease severity could be avoided every year.

In order to answer what is proposed, this trial uses a robust methodology because it is randomized, with hidden allocation, blinded statistical analyst, and used the intention-to-treat approach. Thus, it reduces the influence of selection bias, measurement bias, and other errors in the trial results.

However, this study has limitations. Due to the different nature of the interventions, it will not be possible to blind participants, outcome assessors (self-reported), and therapists. In addition, the measurement of the isometric strength muscle outcome can be influenced by the participant’s learning effect during the test. Finally, the BFR will be determined at rest, and therefore, it will not be possible to guarantee that the amount of restricted blood flow is equal during the exercises, as the hemodynamics is altered due to muscle contraction and the release of substances that act on the blood vessels [84].

## Trial status

The expected start of recruitment of participants is March 2022. Recruitment is expected to continue until March 2023, with a 1-year follow-up to be completed in July 2024. The data analysis is expected to be completed in December 2024.

The authors confirm that all ongoing and related trials for this drug/intervention are registered.

## Supplementary Information

**Additional file 1.** SPIRIT Checklist. SPIRIT checklist completed.

## Data Availability

Datasets generated or analyzed during the current study will be openly available in a repository.

## Abbreviations

OA: Osteoarthritis
BFR: Blood flow restriction
BFRplacebo: Blood flow restriction placebo
LOP: Arterial limb occlusion pressure
BFR40%: 40% of the total arterial limb occlusion pressure
BFR80%: 80% of the total arterial limb occlusion pressure
HIRE: High-intensity resistance exercise
LL: Low load
RM: Repetition maximum
ICF: Informed Consent Form
VAS: Visual analog scale
MMSE: Mini-Mental State Exam
WOMAC: Western Ontario and McMaster Universities Osteoarthritis Index
OARSI: Osteoarthritis Research Society International
TUG: Time Get Up and Go Test
IPAQ: International Physical Activity Questionnaire
SF 36: Short-Form Health Survey
GPE: Global Perceived Effect
